# Antiseptics in Minor Surgery

**Published:** 1900-05-19

**Authors:** Leonard A. Bidwell

**Affiliations:** Senior Assistant Surgeon to the Hospital, and Dean of the Post-Graduate College.


### Hospital Clinics and Medical Progress.

### ANTISEPTICS IN MINOR SURGERY.

A Post-graduate Lecture at the West London Hospital, given by Leonard A. Bidwell, F.R.C.S., Senior Assistant Surgeon to the Hospital, and Dean of the Post-graduate College.

Although the necessity for the employment of antiseptics in all operations of any magnitude is everywhere recognised, many minor procedures and dressings are still conducted in a manner falling far short of an aseptic ideal. I have, therefore, thought it allowable to devote this lecture to some suggestions on the employment of antiseptics in minor surgery. In the first place, the cleanliness of the pocket case of instruments is most important, and to facilitate asepsis in this particular various aseptic pocket cases have been introduced; these are mostly made of German silver or aluminium, and all the contents, including the knives, take to pieces for the purpose of boiling. Of course, this is an ideal sort of case, and when a surgeon has to purchase a new case, such a one should be insisted upon; but, with the exception of the old clasp pocket-knives, the old-fashioned leather pocket cases can be made to answer very well if certain precautions are taken. One word as to the best selection of instruments in a pocket case. The following are the contents of a case which is being made for me by Messrs. Arnold: (1) A small scalpel with metal handle; (2) dissecting forceps with shark's teeth; (3) two pairs of Spencer Wells' artery forceps; (4) straight scissors; (5) small double sharp spoon; (6) steel director, with one end open and an aneurism needle at the other end; (7) probe; (8) metal tube containing needles at one end and a spool of chromic catgut and horsehair at the other; (9) pair of special fine curved dressing forceps. The instruments are arranged in racks, which can be taken out of the case and the two halves of the case can be separated, and be used, one for holding antiseptic lotion, and the other as a tray for the instruments. This case will contain everything necessary for a small operation or for the first dressing of a wound, but it does not contain many of the ordinary articles found in a dressing-case, such as a thermometer, a caustic case, a spatula, and a trocar, since I think that these are best carried separately in the bag or the pocket. One knife is all that can be required, and it will answer every purpose; two or three others are kept at home to replenish the case.

All the steel instruments in a case, whether new or old-fashioned, should be boiled for five minutes in a 1 per cent. soda solution at least twice a week, as well as immediately after use in any septic case. When all the contents of the case have been completely turned out, the case, if a metal one, should also be boiled, and if a leather one, it should be thoroughly wiped all over with a wet carbolic towel. Of equal importance with the instruments are the ligatures to be carried in the pocket case; silk is, in my opinion, the most unsuitable material for the purpose, since it should only be used after being thoroughly sterilised, and even then requires to be kept in an antiseptic solution in order to prevent contamination. The least objectionable and most easily carried ligatures are dry chromic catgut, or the dry sterilised catgut, sold, in sealed aseptic cases, by Seabury and Johnson; either of these only require to be soaked in some antiseptic solution prior to use. The best material for sutures is horsehair and silkworm gut, which can be carried dry, wrapped up in antiseptic gauze, and soaked in antiseptic before use. Horsehair is readily sterilised by soaking or wiping in carbolic or perchloride solution, and it sets up less irritation than any other material, the danger of a scar being produced by the stitch-holes being minimised. Needles, after sterilisation, should be carried in a small glass or metal tube or box.

With regard to dressings, the best, undoubtedly, is the double cyanide gauze; this should not be carried loose in the bag, but should be wrapped up in mackintosh, or, better still, in a sponge-bag, which is kept for this purpose alone; it is best to keep the gauze damped in 1 in 40 carbolic solution. Some antiseptic agent must also be carried, both for the instruments and for bathing the wound. Carbolic is the most suitable for the instruments, and is equally good for the wound; it can be carried in the form of soloids, as introduced by Messrs. Burroughs and Wellcome. Chinosol, also, is harmless to instruments; and this, too, can be carried in the soloid form. Mercury, which is the best for wounds, is, however, very objectionable for instruments, and so is unsuitable if only one antiseptic can be carried.

For the cleansing of the wound wool mops are usually employed, but the use of these immediately after a second's soaking in an antiseptic is not ideal, and it is better to prepare some mops some time beforehand and to carry them damped with antiseptic fluid in another sponge bag, or Bernay's antiseptic compressed wool sponges may be used, and answer the purpose admirably. We now come to the preparation of the surgeon's hands, and of the skin of the patient. I find the simplest way to purify the hands is to wash them thoroughly with a nail brush with a fluid soap. Johnston's ethereal fluid soap is an excellent preparation, as ether dissolves the fat in the entrance to the hair follicles, and so allows the soap to penetrate more deeply than ordinary soaps. After this the hands are soaked in either a 1 in 40 carbolic solution or in 1 in 1,000 mercuric chloride solution. The parts round the wound are prepared in a similar way, but turpentine here is also very useful, and should always be employed in the absence of an ethereal soap.

For a small wound I always fix the dressing with collodion; a narrow strip of three or four layers of gauze, damped in carbolic, is first applied, and over this a much larger piece of a single thickness of dry gauze is placed, and the edges fixed on with collodion; it will be found best to use the ordinary, and not the flexible collodion.

I would urge some such precautions in minor surgery, although we know that most simple wounds will often do well without any such care; but, in order to prevent a surgical disaster, no excess of care can be too great.

